# Review of UK and Ireland Surveys of Health Professional Educators on Teaching of Sexual- and Gender-Minority Health

**DOI:** 10.3390/bs16010075

**Published:** 2026-01-06

**Authors:** Catherine Meads, Christopher Morrison

**Affiliations:** 1 Faculty of Health, Medicine and Social Care, Anglia Ruskin University, Cambridge CB1 1PT, UK; 2 Institute of Education in Healthcare and Medical Sciences, School of Medicine, Medical Sciences and Nutrition, University of Aberdeen, Aberdeen AB24 3FX, UK; christopher.morrison@abdn.ac.uk

**Keywords:** education, sexual orientation, gender identity, LGBTQ+, survey, health professional trainers, university

## Abstract

Sexual and gender minority (SGM) people report considerable dissatisfaction with health services compared to heterosexual and/or cisgender people, with health professionals highlighting insufficient training. Teaching about the health of SGM people is not mandated in UK health professionals’ educational curricula. A review of published and unpublished surveys and of UK training courses evaluating LGBTQ+ content, in the UK and Ireland, examining the teaching of the health of SGM people to health professionals was conducted. Fifteen surveys from the perspectives of educators and students were compared and contrasted. Surveys were found from educators from undergraduate medicine, nursing and midwifery, and pharmacy schools, from students at dental, medical, and pharmacy schools, and from qualified doctors and paramedics. Students and clinical staff perceived that they have insufficient training in SGM health, although there is a contradiction in the perception of teaching amount between students and educational staff. Two curriculum reviews of Royal College postgraduate medical training showed either no or very few requirements on SGM health. Although some courses make considerable efforts to fully incorporate SGM health into mandatory curricula appropriately, professionals mention insufficient training. Until curriculum setters mandate SGM health, patient complaints will continue.

## 1. Introduction

Sexual (lesbian, gay, bisexual) and gender (transgender and non-binary) minorities report considerable dissatisfaction with care provided by UK health services compared to heterosexual and cisgender people. Elliott et al. (2015) found that UK sexual minority people were more likely than heterosexual people to report unfavourable experiences with each of four aspects of primary care (no trust or confidence in the doctor, poor/very poor doctor communication, poor/very poor nurse communication, and fairly/very dissatisfied with care overall). The UK Government Equalities Office (2018) found that, in the 12 months preceding data collection, 40% of transgender respondents and 13% of sexual minority respondents had had at least one negative experience of healthcare because of their gender identity or sexual orientation. Given the mounting health inequalities data being generated (Saunders et al., 2021; Semlyen et al., 2016), the difficulties with accessing healthcare (Elliott et al., 2015; Meads et al., 2019), and the increasing proportions in the population who identify as being of minority sexual orientation and gender identity (Office for National Statistics, 2025; Shrimpton, 2020), there is an increasing need for health professionals to be aware of sexual and gender minority health issues and to be comfortable with providing appropriate care.

In the UK in 2006 the NHS Sexual Orientation and Gender Identity Advisory Group (SOGIAG) published a set of Core Training Standards for Sexual Orientation (Cree & O’Corra, 2006) with the aim to make NHS inclusive for sexual minority people, but the standards were never widely adopted. In 2010 the UK government passed the Equalities Act, which includes the Public Sector Equality Duty, meaning that public bodies such as the NHS must consider all individuals when carrying out their day-to-day work when shaping policy and delivering services (Anon, 2010). In 2014, the Association of American Medical Colleges published guidance on curricular and institutional climate changes to help construct a more LGBT+ inclusive environment for newly qualified doctors, including a set of 30 LGBT+ health competencies (Hollenbach et al., 2014).

Several sources suggest a lack of suitable diversity training for health professionals in the UK (Hunt et al., 2019; Muschialli et al., 2025; Somerville, 2015) and internationally (Bleasdale et al., 2022; Gisondi et al., 2023). Many UK staff still do not consider sexual orientation or gender identity important when delivering services (Berner et al., 2020; Somerville, 2015). This may be mirrored by students in healthcare courses who also think a person’s sexual orientation and gender identity is not relevant when delivering person-centred care. Some of this may be driven by lack of appropriate equality and diversity training for health professionals (Somerville, 2015), and there may be many other factors involved such as strong religious or cultural beliefs (Wahlen et al., 2020). Historically, sexual orientation and health issues tended to be taught within the sexual health part of the curriculum, focusing on the assumption that sexual health is the primary health concern for sexual minority people, or just focusing on human immunodeficiency virus experienced by men who have sex with men (Sekoni et al., 2017), thus ignoring other health inequalities. Trans health tends to focus on transitioning or on gender issues, thus ignoring other health inequalities experienced by trans people (Browne & Lim, 2008; de Blok et al., 2021; Seal, 2019).

One of the first surveys about the inclusion of sexual orientation in healthcare professional curricula was conducted in UK medical and dental schools in 1999 (Bewley & Bolton, 1999). In the USA, a more in-depth survey was conducted in all allopathic and osteopathic medical schools in USA and Canada (Obedin-Maliver et al., 2011). In OECD countries there have been several surveys on medical school training from the perspectives of lecturers and students, with recent publications reviewed by Wynn et al. (2024). There is far less information on the teaching of sexual orientation and gender identity to other health professions.

This research reviews available UK and Irish published and unpublished surveys of health professionals’ training in sexual orientation and gender identity from the perspectives of trainers and students, including curriculum reviews. The aim was to bring together this information in one place and to determine whether any trends could be seen across surveys. Practical recommendations to curriculum developers, course organisers, and lecturing staff about improvements that can and should be made in their courses are made.

## 2. Methods

A review was undertaken of available surveys (published and unpublished, in the UK and Ireland). Included were surveys of any health professionals’ training in sexual orientation and gender identity, from the perspectives of trainers, students, or clinical professionals reporting any element of LGBTQ+ content and their reactions to it. These included surveys in any relevant health professional group, including dentistry, medicine, midwifery, nursing, paramedicine, and pharmacy, where data collection had been conducted from 2015 onwards. Conference abstracts and unpublished manuscripts were included if they reported empirical results. Excluded were single case studies or surveys conducted as part of teaching sessions. We also report on curricula reviews of UK training courses evaluating LGBTQ+ content that were found during the searches.

Examples were identified through networking with members of the LGBTQ+ community within the health professions and the use of contact lists within these networks. LGBTQ+ networks used included a specialist LGBTQ+ health research email list, the contact list for GLADD (The Association of LGBTQ+ Doctors and Dentists), the University Network of LGBT Networks email list, NHS staff LGBT contact lists for various health professional groups, and the NHS England LGBT Health Team. A systematic review on the effectiveness of interventions used in training healthcare professionals to introduce or enhance the teaching of LGBTQ+ health issues (Meads & Morrison, 2023) contributed search results that were also used to identify surveys. These searches were updated in August 2025.

One author assessed eligibility according to the inclusion criteria and extracted relevant data, which were checked by a second, with disagreements resolved through discussion. Results and quality of each study are described narratively and through tabular form.

## 3. Results

Summary characteristics of all included studies can be found in Table 1.

### 3.1. Educational Staff Surveys

Six surveys were found evaluating the teaching of LGBT content in the curriculum via asking educational staff. Three were related to medical schools (Ahluwalia et al., 2024; Meads & Morrison, 2023; Tollemache et al., 2021), one to nursing and midwifery schools (Brown et al., 2021), one to pharmacy schools (Mawdsley & Willis, 2023), and one to plastic surgery and urology training programme directors (Pigeon et al., 2023).

The UK National Institute for Health Research (NIHR) funded an exploration of teaching about minority group health in the undergraduate medical curricula, including sexual orientation and gender identity (Ahluwalia et al., 2024). Their survey had a 29% response rate (16/55 medical schools) and found that sexual and gender minority health emerged as the most frequently addressed topic (identified by 94% of respondents). The primary barriers were time constraints (71%), inadequate resources (64%), and varying levels of educator confidence (36%). Another barrier was lack of LGBTQ+ placements for students.

Meads and Morrison (2023) conducted a survey focusing on dentistry, midwifery, nursing, paramedicine, and pharmacy, and it had a 15% (22/143) response rate. They also attempted to survey postgraduate medical training via Royal Colleges, but none responded. The question set was very similar to that used by Tollemache et al. (2021). Of responders, 55% of courses had an element of sexual and gender minority health content. In some instances, gender identity health training was being given rather than combined sexual orientation and gender identity, leading to the possibility that some students were thinking they had covered LGBTQ+ health when they had only been given some of the training they needed. Some respondents indicated that they considered that material on sexual and gender minority health was unsuitable or not relevant for students.

The Tollemache survey (Tollemache et al., 2021) surveyed all 37 UK medical schools with students enrolled in a primary undergraduate medical training course. They had a 51% response rate and found that nearly all of the medical schools surveyed included some form of sexual and gender minority health content in their curricula. The median estimated LGBT-teaching hours across the entire undergraduate course was 11.0, with an inter-quartile range of 12.25. LGBT mental health, gender identity, sexual orientation, and awareness of specific LGBT health inequalities were the most commonly covered topics.

Brown and colleagues released a report entitled ‘Making the Invisible Visible-The inclusion of sexual and gender minority health needs and concerns within nursing and midwifery pre-registration programmes’ (Brown et al., 2021). This covered UK and Ireland but was conducted during the start of the COVID-19 pandemic and had a response rate of 21% (29/138). They found the following results:

*“Whilst 50% of nursing respondents and 68% of midwifery respondents considered it ‘very important’ to provide content and integrate LGBTQ+ health issues and concerns within the pre-registration programme, only 10% and 6% respectively felt such inclusion was ‘fully adequate’”.*

There was an appreciation that sexual and gender minority content was not explicitly part of Nursing and Midwifery Council education standards, and there was frustration that it was missing. Many respondents stated that sexual and gender minority content was subsumed within equality, diversity, and inclusion themes and not made explicit.

Mawdsley and Willis (2023) surveyed UK university master’s in pharmacy course leads about hetero and/or cisnormativity in their pharmacy curricula. They used the same survey as did the Tollemache et al. (2021) study and had a 62% response rate (18/29). They found that 61% viewed pharmacy curricula as both heteronormative and cisnormative in design. Thirty-seven percent indicated that gender identity was mentioned, and 26% indicated that sexual orientation was mentioned. The most commonly covered topics were HIV/AIDS and sexually transmitted diseases in LGBTQI+ people.

The study evaluating plastic surgery and urology training programme directors (Pigeon et al., 2023) focused on gender affirming care of transgender patients only. Response rates were 79% (11/14 in plastic surgery) and 68% (13/19 in urology), and they estimated that these responses represented 487 trainees. They also estimated that the total exposure of UK trainees to any aspect of gender affirming healthcare was a median of 1 h training per trainee per year (range 0–51 h). Only 24% (8/33) had direct clinical training and 33% (11/33) had no training at all. No training programme directors reported that their trainees had had instruction in masculinising genital surgery.

### 3.2. Student Surveys

One study surveyed dental students (Lennox et al., 2022), four studies surveyed medical students (Arthur et al., 2021; Barber et al., 2023; Eyskens et al., 2020; Parameshwaran et al., 2017), and one study surveyed pharmacy students (Mawdsley & Willis, 2023).

Lennox et al. (2022) surveyed dental students at a single dental school, with a response rate of 20.5% (89/435). The majority of participants (57%) were in the clinical years of their studies. Forty-one percent indicated that they were uninformed about LGBT+ people’s barriers to healthcare, and 13.5% reported that they had felt uncomfortable speaking up on LGBT+ issues because of fear of negatively impacting patient rapport. One-third of these described their sexual orientation as homosexual or bisexual.

Arthur et al. (2021) surveyed medical students in years 1–5 from one medical school in southeast England, using some unique questions and some based on the survey used by Parameshwaran et al. (2017). They had a 32% response rate (252/776) and found positive attitudes towards LGBT patients but deficits in medical students’ confidence and knowledge of their specific health problems. Sixty-nine percent considered that they had not received specific training on LGBT health needs, and 85% wanted more training.

Barber et al. (2023) surveyed medical students at all UK medical schools using a 15-question survey circulated via social media. They received responses from 296 students from 28 UK medical schools. They found that 59.1% of participants reported that they had had no teaching on sexual and gender minority healthcare. Furthermore, only 12.5% of participants felt their knowledge on sexual and gender minority healthcare was sufficient, and 97.2% reported that they wanted more teaching on LGBTQ+ healthcare as part of the medical curriculum. Participants suggested methods of incorporating this training by having access to people with lived experiences of discrimination within healthcare and through the inclusion of sexual and gender minority patients within assessments.

Eyskens et al. (2020) conducted a survey on gender identity issues covered in the curricula of students attending London medical schools in 2020. They received 295 valid responses from students at Barts, Imperial, Guys and Kings, St George’s, and UCL medical schools in years 1–5 and those intercalating. Over three-quarters reported that they had never been taught about trans healthcare specifically and over 80% wanted to have some teaching on trans healthcare within the curriculum.

Parameshwaran et al. (2017) surveyed medical students in years 1–6 from one medical school in southern England, using some unique questions and some based on the survey used by a US study (Sanchez et al., 2006). The majority of students were in their clinical years of study. The survey had an 18% response rate (166/938) and found generally positive attitudes towards LGBT patients. Eighty-five percent of participants ‘strongly disagreed’ or ‘disagreed’ that they had received LGBTQ-specific healthcare training, and most were unconfident regarding LGBTQ-specific healthcare terminology.

Mawdsley and Willis (2023) surveyed pharmacy students as well as teaching staff (as described above). The student survey received 458 responses from an estimated eligible population of 15,635 in 25 universities (2.9% response rate), but only 136 students’ responses were reported. They found that 71% of students viewed pharmacy curricula as both heteronormative and cisnormative in design. Interestingly, far fewer students than staff considered that topics were covered in the curriculum for each of the 22 topics surveyed. For example, 2% of students compared to 37% of staff indicated that gender identity was mentioned in the curriculum, and 2% of students compared to 26% of staff indicated that sexual orientation was mentioned.

### 3.3. Clinical Staff Surveys

Four surveys were found, one in junior medical staff (Shanmugam & Vadeyar, 2021), two in senior medial staff (Berner et al., 2020; Quigley et al., 2023), and one in paramedicine (Gunn, 2019). In addition, two media releases describing a 2016 RCN survey of more than 1200 UK nursing staff about care of trans patients were also found, but we could not obtain access to the survey itself (Royal College of Nursing, 2016). According to the media releases, the study found that 76% had encountered trans people during their healthcare work and 56% had cared for trans people directly. Of those nurses who had directly cared for a trans patient, 87% felt unprepared to meet their patient’s needs. Only one-fifth of all nurses surveyed said they thought the nursing workforce had the skills to care for trans adults and children, while 76% said more training for all healthcare staff was needed.

Shanmugam and Vadeyar (2021) surveyed all foundation doctors in the West Midlands Deanery hospitals to assess the training provided to them in caring for sexual and gender minority patients (e.g., talks given during induction, modules at medical school). They found that, of 60 respondents, only 3% had received any education during their foundation years and that 76.7% felt having formal training in this area would be useful.

Berner et al. (2020) evaluated self-perceived knowledge, attitudes, and behaviours of 238 UK oncologists (65% consultants and 35% registrars) about LGBTQ+ patients with cancer. They estimated that the response rate was ~10%. Most oncologists felt comfortable treating LGBTQ+ patients, but most lacked population-specific cancer knowledge, and many may fail to encourage disclosure of LGBTQ+ status. Sixty-eight percent felt that LGBTQ+ healthcare should be a mandatory component of postgraduate training.

Quigley et al. (2023) surveyed members of the Irish Association of Dermatologists about transgender healthcare, with a response rate of 30% (41/135), of which 44% were consultants and 56% were registrars. Only 10% (4/41) had previously received education around the dermatological care of transgender people; two felt it was sufficient to make them feel confident in the care of transgender patients, and the other two did not. Seventy-five percent of respondents agreed that they would benefit from further education on this topic.

The National Ambulance LGBT Network surveyed their members (N = 443) in 2018 (Gunn, 2019). Almost all the survey was about the experiences of people working in the service and not about training. However, there was one very interesting and relevant comment:

*“The equality training is appalling and generally delivered by heterosexual people. Whilst most people are okay with gay people I have found a lot of staff, whether it be thinking they are doing the right thing, have outed me during patient contact or in conversation. Whilst I have no problem with being gay it is my choice who I tell and being a bit older growing up around Section 28 have done this historically for my personal safety. There seems to be a big misunderstanding around this in terms of confidentiality.”*

### 3.4. Curriculum Content Evaluations

Two medicine-related curriculum content evaluations were found (Lundrigan, 2025; Swift, 2019). Lundrigan (2025) identified the extent of inclusion of LGBTQ+ health in the medical curricula of the General Medical Council (GMC) standards for medical education and training (undergraduate and postgraduate) and the 24 Colleges in the Association of Medical Royal Colleges (AOMRC). She found that six curricula were inaccessible. The GMC standards and 85% (15/18) of the accessible AOMRC curricula had no mention of LGBTQ+ content. Moreover, the three that did have some form of inclusion had massive gaps in terms of actual coverage. For example, the Faculty of Sexual and Reproductive Health curriculum included a section on ‘Managing transgender health problems’, which also has mention of non-binary individuals but no mention of sexual orientation.

Swift (2019) conducted a master’s degree dissertation on an LGBT+ content analysis of a random selection of Royal College and Faculty curricula, including those for general practice, obstetrics and gynaecology, psychiatry, sexual health, and the GMC general requirements for postgraduates (Swift, 2019). She found no mention of the terms LGBT, gay, MSM (men who have sex with men), lesbian, bisexual, transgender, non-binary, sexuality, or sexual orientation in the GMC general requirements. There was one mention of sexual orientation in the child psychiatry curriculum but nothing at all in the core and general psychiatry curriculum. The Royal College of General Practice curriculum had only one mention of gay, lesbian, and transgender. The Royal College of Obstetricians and Gynaecologists’ curricula mentioned transgender and non-binary six times, with one mention of sexuality. The Faculty of Sexual and Reproductive Health curriculum had multiple mentions of LGBT, MSM, lesbian, transgender, sexuality, and sexual orientation. The curriculum for genito-urinary medicine (from the Joint Royal Colleges of Physicians Training Board) had multiple mentions of MSM, bisexual, transgender, non-binary, and sexuality. These terms were mostly used in the context of heteronormativity, grouping LGBT+ people with medical conditions such as HIV, and resulted in invisibility, such as no mention of lesbians in the Royal College of Obstetricians and Gynaecologists’ curricula.

## 4. Discussion

We found 15 surveys and two curriculum reviews on the training of UK health professionals in LGBTQ+ health. There is reasonably good evidence from two surveys (Ahluwalia et al., 2024; Tollemache et al., 2021) that most UK undergraduate medicine courses have some element of LGBTQ+ health training in their curricula, but the same cannot be said for other health professional courses (Brown et al., 2021; Mawdsley & Willis, 2023; Meads & Morrison, 2023). Far less information is available about the content of courses in nursing, midwifery, and pharmacy from the perspectives of educational staff, and nothing is available about dentistry, paramedicine, or of the curricula designed by the UK Faculties and Royal Colleges responsible for postgraduate medical training. More worryingly, student survey results suggest that students are somehow missing this content and feel they are insufficiently trained to meet the needs of the LGBTQ+ patients they will encounter. Clinical staff surveys suggested a continued lack of LGBTQ+-specific health knowledge and a lack of training for most staff, yet there is an increasing confidence with increasing seniority. Curriculum reviews of the Medical Royal Colleges show most have no mention of required LGBTQ+ content for their trainees, when these are supposed to be trained specialists. The survey of trainees expected to perform gender affirming care of transgender patients (Pigeon et al., 2023) showed that most had no training in the majority of functions they may be expected to undertake.

It is possible that many health professionals currently receive insufficient training in sexual and gender minority issues during their undergraduate or pre-qualification courses, or during their postgraduate courses or post-qualification portfolio development. Where they do receive some training, it is probably inadequate for many health professionals. Reasons why so many courses have not incorporated sufficient sexual and gender minority health issues include the following: lack of relevant experience within the faculty, lack of perceived relevance to the students, insufficient funding available, lack of space or time constraints within the curriculum, insufficient knowledge about the implementation of sexual and gender minority health-inclusive teaching, and lack of perceived importance. A few medical schools and other health professional courses have made considerable efforts to fully incorporate sexual and gender minority health into their mandatory curricula appropriately (Salkind et al., 2019), and much could be learned from their efforts. Some of these have been student-led, and some were staff-led or a combination.

### 4.1. Strengths and Weaknesses

One of the major strengths of this project is using a review to fill gaps in knowledge on LGBTQ+ health teaching to health professional students from the perspectives of staff and of undergraduate and postgraduate students. The review included unpublished work, indicating that extensive contact with the LGBTQ+ health community was successful.

One issue with this kind of review is that each of the surveys asked slightly different questions. We have attempted to report what they actually asked, but that means the issues highlighted will not always be concurrent across the included studies. Another major weakness in many surveys is low response rates, despite numerous efforts by researchers. Part of the low response rates in this review may have been associated with the COVID-19 pandemic and that numerous university staff were extremely busy with coping with changes in teaching methods from face-to-face to online teaching and back again, as social distancing rules changed over the course of survey data collection periods. It is possible that some people who completed the surveys were either from the sexual or gender minority themselves or strong allies to the community, so they understood why these surveys were being conducted. This may have biased the responses obtained, but as most surveys did not ask about the demographics of the education staff completing the survey, there was no real way of knowing this. There are other forms of bias that may have occurred within each of the surveys, and none addressed or accounted for bias, but some discussed the implications of several types of potential biases. However, despite the low response rates and biases, the consistency of findings across surveys demonstrates that these findings are likely to be robust. It is never possible to know whether there were any surveys that were missed from our review.

It was not possible to distinguish between token coverage versus integrated, comprehensive training. Without close examination of the curriculum of each establishment and how it was operationalised in lectures, seminars, and other teaching materials, it would not be possible to determine appropriateness of the material covered. However, this issue might be one explanation of why more educators considered that LGBTQ+ topics were covered than students did.

### 4.2. Implications for Policymakers

Members of the sexual and gender minority community make up at least 5% of the population (Geary et al., 2018) and are likely to increase (Office for National Statistics, 2025). Evidence from UK patient surveys shows that, on average, sexual and gender minority patients experience more unfavourable episodes of care, have less trust or confidence in doctors, poor or very poor doctor and nurse communication, and more dissatisfaction with care overall compared to heterosexual or cisgender patients. There are numerous calls from students and junior staff to learn about sexual and gender minority health issues. A UK systematic review on unconscious bias in the curriculum (Borkin, 2021) found that, for medical schools, the lack of training in the full range of protected characteristics meant that students were often ill-equipped to treat the patients in front of them.

Several high-level reports have indicated that health professionals should have some element of sexual and gender minority health in the curriculum (Cass, 2024, p. 212; Women and Equalities Select Committee, 2019, p. 26). However, professional regulators such as the General Medical Council, the Nursing and Midwifery Council, and the Health and Care Professions Council are not explicit around the need for incorporating sexual and gender minority issues into curricula. Their learning objectives tend to be much more generic around the need for students to understand about equality and diversity. Therefore, course planners in individual universities are not obliged to incorporate sexual and gender minority health issues into the curriculum.

There is no universally agreed curriculum or learning objectives on sexual and gender minority health appropriate for each profession. There is very little exploration currently as to whether the training that is being delivered is what learners require. Our assessment of developments in this area suggests that there is a spectrum of activity:

- No input;
- Negative stereotyping, e.g., the only example in the curriculum is a gay man with a sexually transmitted infection;
- A one-off lecture, frequently given by activists (student or staff);
- A series of lectures or training events;
- Incorporation of scenarios on sexual and gender minority health issues;
- Incorporation of sexual and gender minority people into scenarios on other health-related training;
- Incorporation on sexual and gender minority health issues into exams and assessments.

Where one-off lectures are being used, they are usually from a student or staff activist with a link to that course, do not have a regular slot in the curriculum and, may be lost as soon as the student or staff member leaves the institution. These one-off lectures tend to be of variable quality, sometimes focus on just gender identity and trans issues rather than both sexual and gender minority health, have not been peer reviewed, and are rarely evaluated. Once a series of lectures is planned, it tends to be more likely to have a regular slot in the curriculum, tends to have a planning committee of various interested parties, and tends to be more integrated in the overall teaching of the course. Where scenarios are being used, they can either be explicitly around sexual or gender minority health, or be on other health issues which happen to also have a sexual or gender minority aspect, such as a scenario around delayed presentation of cervical cancer in a lesbian who had not attended screening, so students learn both about cervical cancer and reasons why some populations do not participate in screening. When sexual and gender minority health materials occur in exams and other assessments such as OSCEs, it can be assumed that the course has more or less fully integrated sexual and gender minority health issues into the curriculum. There are currently no specific UK-based training-the-trainer courses on sexual and gender minority health for health professional education staff in any of the professions. Given the likely increase in LGBT patients over time, if training does not improve, there will be more unfavourable episodes of care, even less trust or confidence in doctors, and even more dissatisfaction with care.

### 4.3. Implications for Research

Essential aspects to consider are what is meant by sexual and gender minority health issues and what aspects of these could be usefully introduced into the curricula of each type of health professional. This is not a new branch of medicine but basic information that all health professionals need to know. However, if discussions of sexual minority people and their health are introduced into the mental health curriculum, the subtext is that sexual identities other than heterosexuality is a mental illness, which is incorrect. If gender identity is introduced into the communication skills module, the subtext is that once NHS staff understand how to communicate with trans patients, then all will be well, which is untrue. It is currently unclear how, where, and what should be introduced. Research could be conducted regionally taking a cluster approach, where some areas or regions lead with the introduction of sexual and gender minority health materials into the curriculum straight away, using the information already available, whereas others start by using a more measured approach and piloting the introduction to evaluate which types of training are more useful, acceptable, and efficient for improving students’ learning. A randomized controlled trial of the effectiveness of a sexual and gender minority health education intervention should be conducted to improve the evidence base and measure student success with relevant examinations. Any work around the introduction of sexual and gender minority health issues should include patient and public involvement from the LGBTQ+ community. Ultimately, the health of sexual and gender minority patients should be evaluated as part of any of these studies.

As influential policy recommendations on education of professionals in sexual and gender minority health have not yet been acted on, it is unclear what will succeed in incorporating this topic into curricula. Research to address this would include understanding which attitudinal and behaviour aspects are limiting change, and to develop practical solutions. The Behaviour Change Wheel (Michie et al., 2014) is a well-established set of methods for designing interventions that are intended to change behaviour, and this could be used to establish the required solutions.

## 5. Conclusions

There is some evidence in undergraduate and postgraduate medicine, nursing, and midwifery that some courses cover LGBTQ+ health, but it is patchy, inconsistent, and may not cover what is needed by students. There is no evidence in Medical Royal College curricula. Until curriculum setters specify sexual and gender minority health, most health professionals will not gain sufficient training in this area, and patient complaints and health inequalities will continue.

## Figures and Tables

**Table 1:** Included survey characteristics.

Study, Date (Location)	Type of Output	Discipline	Respondent Category	Postgraduate/ Undergraduate	Response Rate
Ahluwalia et al. (2024) (UK)	Report on website	Medical school	University educational staff	Undergraduate	29% (16/55)
Arthur et al. (2021) (Brighton, UK)	Published journal article	Medicine	Students	Undergraduate	32% (252/776)
Barber et al. (2023) (UK)	Published journal article	Medicine	Students	Undergraduate	Unclear
Berner et al. (2020) (UK)	Published journal article	Medicine	Consultants and registrars	Postgraduate	~10%
Brown et al. (2021) (UK and Ireland)	Report on website	Nursing and midwifery	University educational staff	Undergraduate	21% (14/67)
Eyskens et al. (2020) (London, UK)	Unpublished manuscript	Medicine	Students	Undergraduate	Unclear
Gunn (2019) (UK)	Report on website	Paramedicine	Members of LGBT staff group	Postgraduate	Unclear
Lennox et al. (2022) (Newcastle, UK)	Published journal article	Dentistry	Undergraduate students	Undergraduate	20.5% (89/435)
Mawdsley and Willis (2023) (UK)	Published journal article	Pharmacy	University educational staff and students	Undergraduate	Staff: 62% response rate (18/29) Students: 2.9% (458/15,635)
Meads and Morrison (2023) (England)	Report on website	Medical school	University educational staff	Undergraduate	15% (22/143)
Parameshwaran et al. (2017) (Oxford, UK)	Published journal article	Medicine	Students	Undergraduate	18% (166/938)
Pigeon et al. (2023) (UK)	Published journal article	Plastic surgery and urology	Training programme directors	Postgraduate	79% (11/14 plastic surgery) 68% (13/19 urology)
Quigley et al. (2023) (Ireland)	Published journal article	Medicine	Consultants and registrars	Postgraduate	30% (41/135)
Shanmugam and Vadeyar (2021) (West Midlands)	Conference poster	Medicine	Junior medical staff	Postgraduate	Unclear
Tollemache et al. (2021) (UK)	Published journal article	Medicine	University educational staff	Undergraduate	51% (19/37)

## Data Availability

No new data were created or analysed in this study. Data sharing is not applicable to this article.
